# Census Returns-Physicians’ Registers

**Published:** 1889-07

**Authors:** 


### EDITORIALS AND MISCELLANEOUS.

Dr. Robt. L. Porter, Superintendent of Census returns is sending out to the medical profession throughout the country "Physician's Registers" for the purpose of obtaining more accurate returns of deaths than it is possible for the enumerators to make. It is earnestly hoped that physicians in every part of the country will co-operate with the Census Office in this important work. The record should be kept from June 1, 1889, to May 31, 1890. Nearly 26,000 of these registration books were filled up and returned to the office in 1880, and nearly all of them used for statistical purposes. It is hoped that double this number will be obtained for the Eleventh Census.

Physicians not receiving Registers can obtan them by sending their names and addresses to the Census Office, and, with the Register, an official envelope which requires no stamp, will be provided for their return to Washington.

If all medical and surgical practitioners throughout the country will lend their aid, the mortality and vital statistics of the Eleventh Census will be more comprehensive and complete than they have ever been. Every physician should take a personal pride in having this report as full and accurate as it is possible to make it.

It is hereby promised that all information obtained through this source shall be strictly confidential. Address

ROBT. L. PORTER, SUPERINTENDENT OF CENSUS.

